# Synthesis and comparative structural study of 2-(pyridin-2-yl)-1*H*-perimidine and its mono- and di-*N*-methyl­ated analogues

**DOI:** 10.1107/S205698902100013X

**Published:** 2021-01-08

**Authors:** Paulina Kalle, Sergei V. Tatarin, Alexander Yu. Zakharov, Marina A. Kiseleva, Stanislav I. Bezzubov

**Affiliations:** a Department of Chemistry, Lomonosov Moscow State University, Lenin’s Hills, 1-3, Moscow, 119991, Russian Federation; b N.S. Kurnakov Institute of General and Inorganic Chemistry, Russian Academy of Sciences, Leninsky pr. 31, Moscow 119991, Russian Federation

**Keywords:** crystal structure, perimidine, π–π stacking, hydrogen-bonding, NMR study

## Abstract

The title compounds, 2-(pyridin-2-yl)-1*H*-perimidine (C_16_H_11_N_3_; **1**), 1-methyl-2-(pyridin-2-yl)-1*H*-perimidine (C_17_H_13_N_3_; **2**), and 1,3-dimethyl-2-(pyridin-2-yl)-1*H*-perimidinium iodide (C_18_H_16_N_3_^+^·I^−^; **3**) were synthesized under mild conditions and their structures were determined by ^1^H NMR spectroscopy and single-crystal X-ray analysis. The *N*-methyl­ation of the nitro­gen atom(*s*) at the perimidine moiety results in a significant increase of the inter­plane angle between the pyridin-2-yl ring and the perimidine system. The unsubstituted perimidine (**1**) forms a weak intra­molecular N—H⋯N bond that consolidates the mol­ecular conformation. In the crystal structures of **1**–**3**, the mol­ecular entities all are assembled through π–π and C—H⋯π inter­actions.

## Abstract 2

A series of unsubstituted, mono- and di-*N*-methyl­ated perimidines were prepared and studied by single-crystal X-ray analysis and ^1^H NMR spectroscopy.

## Chemical context   

Perimidines are fused nitro­gen heterocyclic aromatics possessing equally a π-electron excess and a π-electron deficiency that determine their diverse reactivities as well as their unique optical and spectroscopic properties (Pozharskii *et al.*, 2020 ▸). These compounds have attracted considerable attention over the past two decades because of their growing application in industrial chemistry (especially as dyes and pigments), as optoelectronics, in biotechnology and medicinal chemistry (Sahiba & Agarwal, 2020 ▸).
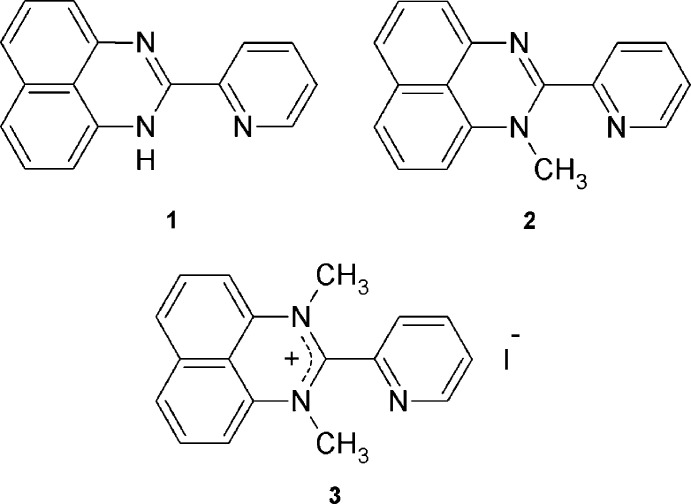


Herein, we report structural studies of 1-*H*-2-(pyridin-2-yl)perimidine (**1**) and its mono- and di-*N*-methyl­ated analogues (**2** and **3**, respectively).

## Structural commentary   

**Figure 1:**
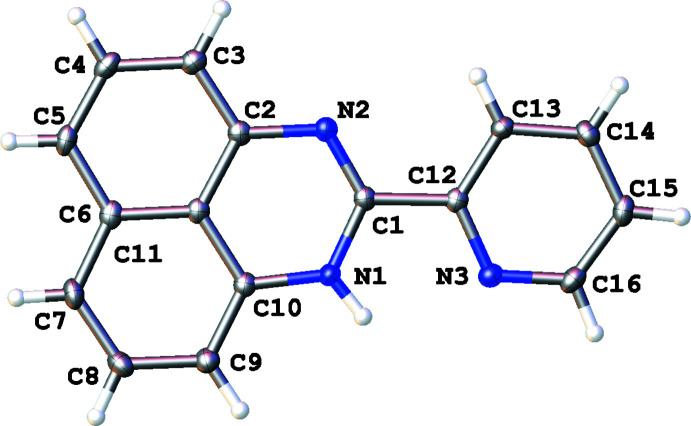
The mol­ecular structure of 1-*H*-2-(pyridin-2-yl)perimidine (**1**), with displacement ellipsoids drawn at the 50% probability level.

The compositions and structures of the synthesized compounds were determined both by ^1^H NMR spectroscopy (for assignment, see: Figs. S1–S3 in the supporting information) and single-crystal X-ray analysis. In all cases, the organic mol­ecules occupy general positions and comprise an essentially flat perimidine system and the pyridyl ring. Depending on the number of *N*-substituents, the ring systems are twisted to a greater or lesser extent (Figs. 1 ▸–3 ▸ ▸). The unsubstituted mol­ecule of **1** is almost planar with the dihedral angle between the aromatic parts as small as 1.60 (5)°, while the mol­ecules of **2** and especially **3** show notably larger inter­plane angles [59.39 (8) and 87.21 (9)°, respectively] because of steric repulsion between the *N*-methyl group(s) and the pyridin-2-yl ring. The flat conformation of **1** may be stabilized by a weak intra­molecular hydrogen bond between the perimidine N1—H1 donor group and the pyridyl N3 acceptor group [*d*(N1⋯N3) = 2.626 (2) Å, *d*(N1—H1) = 0.87 (2) Å, *d*(H1⋯N3) = 2.19 (2) Å, N1—H1⋯N3 = 110.9 (17)°] whereas in the mol­ecular structure of **2** the pyridyl nitro­gen atom participates in a weak intra­molecular C12(*sp* ^3^)—H12*A*⋯N3 contact [*d*(H12*A*⋯N3) = 2.46 (2) Å; C12⋯N3 = 3.059 (1) Å; C12—H12*A*⋯N3 = 120.8 (18)°]. Compound **3** is a salt and its crystal consists of doubly N-methyl­ated perimidinium cations and iodide counter-ions combined mainly through Coulombic inter­actions.

^1^H NMR spectroscopic studies of **1**–**3** revealed correlations between the chemical shifts of some bands in the spectra and the mutual arrangement of the perimidine and pyridyl aromatics. In the ^1^H NMR spectrum of **1** in CDCl_3_, doublets at 6.36 and 6.91 ppm arise from the **j** and **e** protons, respectively, while the other protons of the perimidine core appear as complex multiplets in the range 7.06–7.25 ppm (Fig. S1). A similar set of bands (corresponding to the same protons) with slightly different chemical shifts can be found in the ^1^H NMR spectrum of **2** (Fig. S2) whereas 1,3-dimethyl-2-(pyridin-2-yl)perimidinium iodide (**3**) demonstrates a reduced number of resonance signals (Fig. S3) because the protons of the fused benzene rings become equivalent. The latter results from the above arrangement of the pyridyl ring almost orthogonal to the perimidine system.

For compound **1**, solvent-dependent resonance signals in the ^1^H NMR spectrum were detected. In DMSO-*d*_6_ as a solvent (Fig. S4), the characteristic doublets arising from the protons **j** and **e** are now closer (6.74 and 6.79 ppm, respectively) while the integrated intensity of the signal of the N—H proton becomes lower (0.77 ppm) which may result from a weakening of the intra­molecular N—H⋯N hydrogen bond by the polar solvent.

## Supra­molecular features   

**Figure 4:**
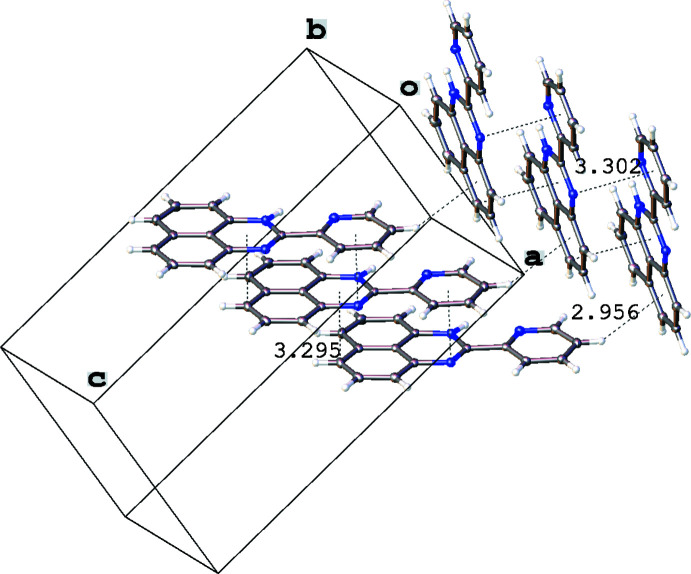
Inter­molecular contacts (Å) in the crystal of 1-*H*-2-(pyridin-2-yl)perimidine (**1**). Displacement ellipsoids are shown at the 50% probability level.

**Figure 5:**
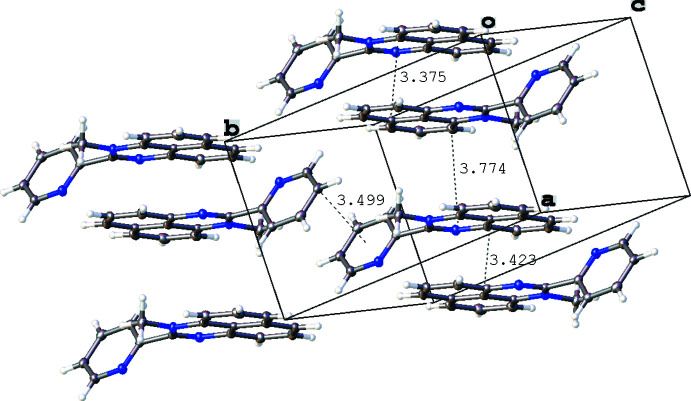
Inter­molecular contacts (Å) in the crystal of 1-methyl-2-(pyridin-2-yl)perimidine (**2**). Displacement ellipsoids are shown at the 50% probability level.

**Figure 6:**
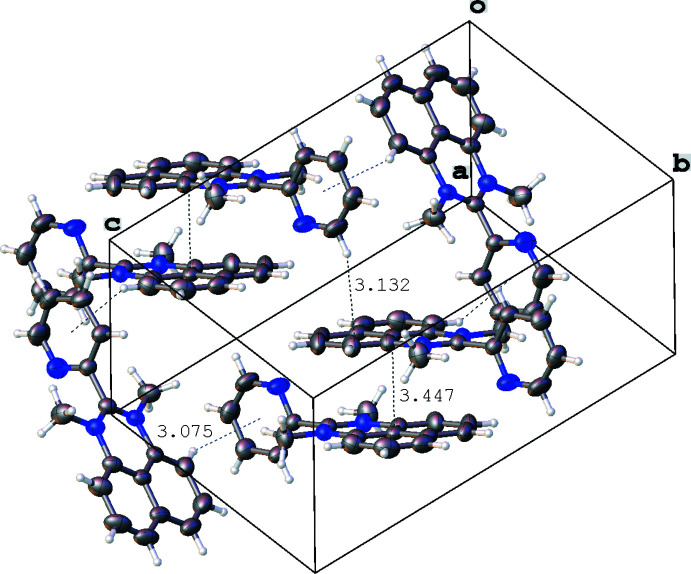
Inter­molecular contacts (Å) in the crystal of 1,3-dimethyl-2-(pyridin-2-yl)perimidinium iodide (**3**, only cations are presented). Displacement ellipsoids are shown at the 50% probability level.

In the crystal of **1**, mol­ecules are assembled through parallel displaced π–π stacking inter­actions between the flat pyridyl and perimidine fragments distant by 3.295 (4) Å (C5⋯N1–C11_centroid_) and 3.302 (4) Å (N2⋯py_centroid_), while the resulting offset stacks [centroid-to-centroid shift between the adjacent mol­ecules in the stack 3.791 (4) Å] are grafted together in the resulting three-dimensional network by a C—H⋯π inter­action [*d*(H⋯π) = 2.96 (2) Å] involving the pyridyl H15 atom and the centroid of the C2–C11 ring (Fig. 4 ▸). In contrast, two types of π–π inter­actions are found in the crystal of **2**, one of which is a slipped stacking [centroid-to-centroid shift 1.645 (2) Å] between the perimidine units [*d*(C7⋯N1–C11_centroid_) = 3.375 (2) Å, *d*(C9⋯N1–C11_centroid_) = 3.774 (3) Å, *d*(C11⋯C6–C11_centroid_) = 3.423 (2) Å] while the other is a pyrid­yl–pyridyl contact [distance between the C16 atom and the pyridyl ring 3.499 (3) Å] connecting the stacks together. Inter­molecular contacts between the H12*C* atom and the C6–C11_centroid_ [3.17 (2) Å] and between the H14 atom and C2–C11_centroid_ [3.684 (19) Å] form a three-dimensional network in the crystal structure of **2** (Fig. 5 ▸). In the crystal structure of **3**, there are π–π-bonded dimers [inter­plane distance 3.447 (3) Å between the perimidine moieties], which form dense layers *via* C—H⋯π inter­actions [*d*(H⋯π) = 3.132 (2) Å between the H18 atom and the centroid of the C6–C11 ring and 3.075 (2) Å between the H9 atom and the centroid of the pyridyl ring; Fig. 6 ▸]. The resulting cationic organic layers and anionic iodide layers alternate along the *c* axis.

## Database survey   

Though many perimidines have been prepared so far, fewer than 60 crystal structures of them (including a few of metal complexes) have been published (Pozharskii *et al.*, 2020 ▸; Hill *et al.*, 2018 ▸; Bahena *et al.*, 2019 ▸; Booysen *et al.*, 2016 ▸). Crystal structures of several 1,3-dimethyl-2-aryl­perimidinium iodides have been determined (Li *et al.*, 2017 ▸). A comprehensive structural study of 2-aryl­perimidines (including those having intra­molecular hydrogen bonds) has also been conducted (Foces-Foces *et al.*, 1993 ▸; Llamas-Saiz *et al.*, 1995 ▸).

## Synthesis and crystallization   

The title compounds were prepared as follows:

1-*H*-2-(pyridin-2-yl)perimidine (**1**).

A mixture of 1,8-di­aminona­phthalene (4.523 g, 28.6 mmol), pyridin-2-ylcarboxaldehyde (2.72 ml, 28.6 mmol) and sodium metabisulfite (16.317 g, 85.8 mmol) in ethanol (50 ml) was refluxed under Ar for 4 h. The reaction mixture was evaporated to dryness, washed with water and redissolved in ethanol. Keeping the resulting solution in a freezer overnight gave a red powder, which was recrystallized from methyl­ene chloride and dried *in vacuo*. Yield 6 g (86%). Single crystals suitable for X-ray analysis were grown by slow evaporation of the solvent from a solution of the substance in methyl­ene chloride.

^1^H NMR (CDCl_3_, ppm, 400 MHz): *δ* 6.36 (*d*, *J* = 7.4 Hz, 1H, H_naph_), 6.91 (*d*, *J* = 7.4 Hz, 1H, H_naph_), 7.06–7.25 (*m*, 4H, H_naph_), 7.44–7.47 (*m*, 1H, H_py_), 7.88 (*td*, *J_1_* = 7.8 Hz, *J_2_* = 1.7 Hz, 1H, H_py_), 8.44 (*d*, *J* = 7.6 Hz, 1H, H_py_), 8.62–8.64 (*m*, 1H, H_py_), 9.39 (*br. s*, 1H, N-H). See supplementary Fig. S1.

1-Methyl-2-(pyridin-2-yl)perimidine (**2**).

To a mixture of **1** (0.250 g, 1.02 mmol), solid KOH (0.057 g, 1.02 mmol) and anhydrous K_2_CO_3_ (0.141 g, 1.02 mmol) in anhydrous Ar-saturated aceto­nitrile methyl iodide (0.064 ml, 1.02 mmol) was added dropwise upon stirring and the resulting suspension was heated at 323 K for 3 h and then at r.t. for two days. The reaction mixture was evaporated to dryness and the crude product was purified by column chromatography (eluent hexa­ne/ethyl acetate 1/1 *v*/*v*), recrystallized from a mixture of CH_2_Cl_2_/hexane and dried *in vacuo*. Yield 185 mg (70%). Single crystals suitable for X-ray analysis were grown by slow evaporation of the solvent from a solution of the substance in chloro­form.

^1^H NMR (CDCl_3_, ppm, 400 MHz): *δ* 3.17 (*s*, 3H, N—CH_3_), 6.32 (*dd*, *J_1_* = 7.2 Hz, *J_2_* = 1.0 Hz, 1H, H_naph_), 6.94 (*dd*, *J_1_* = 7.3 Hz, *J_2_* = 1.0 Hz, 1H, H_naph_), 7.17–7.32 (*m*, 4H, H_naph_), 7.39–7.42 (*m*, 1H, H_py_), 7.77–7.80 (*m*, 1H, H_py_), 7.86–7.89 (*m*, 1H, H_py_), 8.70 (*m*, 1H, H_py_). See supplementary Fig. S2.

1,3-Dimethyl-2-(pyridin-2-yl)perimidinium iodide (**3**).

This compound was isolated from the above reaction mixture (synthesis of compound **2**) as a side product (15 mg). Single crystals suitable for X-ray analysis were grown by slow evaporation of the solvent from a solution of the substance in ethanol.

^1^H NMR (CDCl_3_, ppm, 400 MHz): *δ* 3.34 (*s*, 6H, N—CH_3_), 6.96 (*d*, *J* = 7.7 Hz, 2H, H_naph_), 7.50 (*m*, 2H, H_naph_), 7.60 (*m*, 2H, H_naph_), 7.66–7.70 (*m*, 1H, H_py_), 8.19 (*td*, *J_1_* = 7.8 Hz, *J_2_* = 1.7 Hz, 1H, H_py_), 8.65–8.68 (*m*, 1H, H_py_), 9.24–9.26 (*m*, 1H, H_py_). See supplementary Fig. S3.

## Refinement   

Crystal data, data collection and structure refinement details are summarized in Table 1 ▸. Hydrogen atoms in the structures of **1** and **2** were located from difference electron density maps and were refined freely. In the structure of **3**, hydrogen atoms were placed in calculated positions and refined using a riding model [C—H = 0.94–0.97 Å with *U*_iso_(H) = 1.2–1.5*U*_eq_(C)].

## Supplementary Material

Crystal structure: contains datablock(s) 1, 2, 3. DOI: 10.1107/S205698902100013X/wm5594sup1.cif

Structure factors: contains datablock(s) 1. DOI: 10.1107/S205698902100013X/wm55941sup2.hkl

Supporting information file. DOI: 10.1107/S205698902100013X/wm55941sup5.mol

Structure factors: contains datablock(s) 2. DOI: 10.1107/S205698902100013X/wm55942sup3.hkl

Supporting information file. DOI: 10.1107/S205698902100013X/wm55942sup6.mol

Structure factors: contains datablock(s) 3. DOI: 10.1107/S205698902100013X/wm55943sup4.hkl

Supporting information file. DOI: 10.1107/S205698902100013X/wm55943sup7.mol

Supporting information file. DOI: 10.1107/S205698902100013X/wm55941sup8.cml

Supporting information file. DOI: 10.1107/S205698902100013X/wm55942sup9.cml

Supporting information file. DOI: 10.1107/S205698902100013X/wm55943sup10.cml

Fig. S1. NMR spectrum for 1. DOI: 10.1107/S205698902100013X/wm5594sup11.tif

Fig. S2. NMR spectrum for 2. DOI: 10.1107/S205698902100013X/wm5594sup12.tif

Fig. S3. NMR spectrum for 3. DOI: 10.1107/S205698902100013X/wm5594sup13.tif

Fig. S4. NMR spectrum for 1 in DMSO solvent. DOI: 10.1107/S205698902100013X/wm5594sup14.tif

CCDC references: 2051714, 2032890, 2032889

Additional supporting information:  crystallographic information; 3D view; checkCIF report

## Figures and Tables

**Figure 2:**
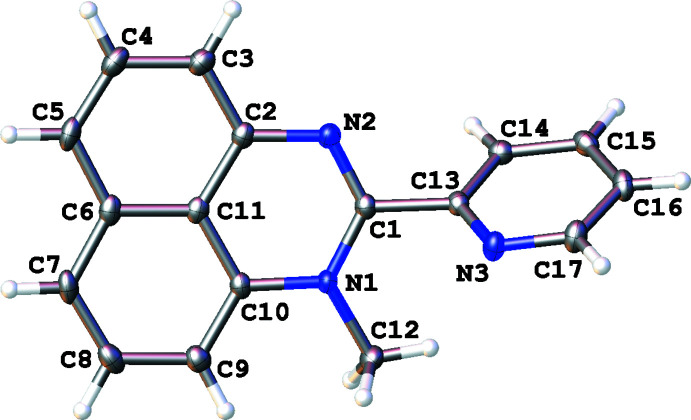
The mol­ecular structure of 1-methyl-2-(pyridin-2-yl)perimidine (**2**), with displacement ellipsoids drawn at the 50% probability level.

**Figure 3:**
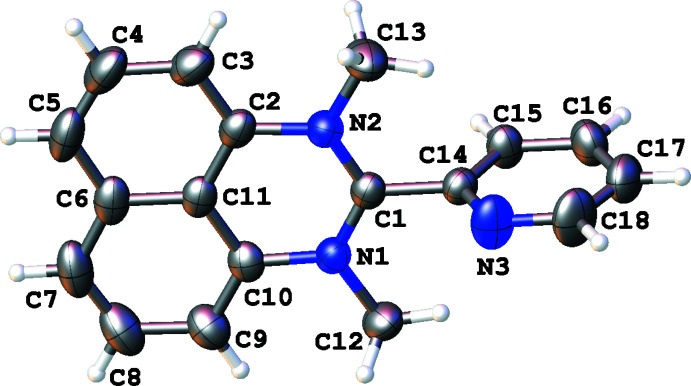
The mol­ecular structure of 1,3-dimethyl-2-(pyridin-2-yl)perimidinium iodide (**3**, only the cation is presented), with displacement ellipsoids drawn at the 50% probability level.

**Table 1:** Experimental details

	(1)	(2)	(3)
Crystal data			
Chemical formula	C_16_H_11_N_3_	C_17_H_13_N_3_	C_18_H_16_N_3_^+^·I^−^
*M*_r_	245.28	259.30	401.24
Crystal system, space group	Monoclinic, *P*2_1_/*c*	Monoclinic, *P*2_1_/*c*	Monoclinic, *P*2_1_/*n*
Temperature (K)	100	100	230
*a*, *b*, *c* (Å)	13.5479 (5), 5.0242 (2), 17.3881 (7)	7.5095 (2), 12.1216 (3), 13.5616 (4)	9.8821 (2), 9.7125 (2), 17.9839 (4)
β (°)	101.382 (2)	92.547 (1)	103.676 (1)
*V* (Å^3^)	1160.28 (8)	1233.25 (6)	1677.15 (6)
*Z*	4	4	4
Radiation type	Mo *K*α	Mo *K*α	Mo *K*α
μ (mm^−1^)	0.09	0.09	1.91
Crystal size (mm)	0.42 × 0.1 × 0.08	0.34 × 0.12 × 0.11	0.32 × 0.18 × 0.13

Data collection			
Diffractometer	Bruker D8 Venture	Bruker D8 Venture	Bruker SMART APEXII
Absorption correction	Multi-scan (*SADABS*; Krause *et al.*, 2015 ▸)	Multi-scan (*SADABS*; Krause *et al.*, 2015 ▸)	Multi-scan (*SADABS*; Krause *et al.*, 2015 ▸)
*T*_min_, *T*_max_	0.684, 0.746	0.685, 0.746	0.668, 0.746
No. of measured, independent and observed [*I* > 2σ(*I*)] reflections	16322, 2866, 2405	14029, 3280, 2803	28389, 4146, 3700
*R*_int_	0.036	0.035	0.026
(sin θ/λ)_max_ (Å^−1^)	0.667	0.682	0.668

Refinement			
*R*[*F* ^2^ > 2σ(*F* ^2^)], *wR*(*F* ^2^), *S*	0.057, 0.140, 1.05	0.060, 0.146, 1.04	0.028, 0.067, 1.06
No. of reflections	2866	3280	4146
No. of parameters	216	233	201
H-atom treatment	All H-atom parameters refined	All H-atom parameters refined	H-atom parameters constrained
Δρ_max_, Δρ_min_ (e Å^−3^)	0.39, −0.33	0.45, −0.34	0.72, −0.46

Computer programs: *APEX3* and *SAINT* (Bruker, 2017 ▸), *SHELXT* (Sheldrick, 2015*a* ▸), *SHELXL* (Sheldrick, 2015*b* ▸), *OLEX2* (Dolomanov *et al.*, 2009 ▸) and *publCIF* (Westrip, 2010 ▸).

## supplementary crystallographic information

### 2-(Pyridin-2-yl)-1H-perimidine (1) Crystal data

**Table:**

C_16_H_11_N_3_	*F*(000) = 512
*M**_r_* = 245.28	*D*_x_ = 1.404 Mg m^−^^3^
Monoclinic, *P*2_1_/*c*	Mo *K*α radiation, λ = 0.71073 Å
*a* = 13.5479 (5) Å	Cell parameters from 7163 reflections
*b* = 5.0242 (2) Å	θ = 3.1–28.3°
*c* = 17.3881 (7) Å	µ = 0.09 mm^−^^1^
β = 101.382 (2)°	*T* = 100 K
*V* = 1160.28 (8) Å^3^	Needle, red
*Z* = 4	0.42 × 0.1 × 0.08 mm

### 2-(Pyridin-2-yl)-1H-perimidine (1) Data collection

**Table:**

Bruker D8 Venture diffractometer	2866 independent reflections
Radiation source: microfocus sealed X-ray tube, Incoatec IµS microsource	2405 reflections with *I* > 2σ(*I*)
Focusing mirrors monochromator	*R*_int_ = 0.036
Detector resolution: 10.4 pixels mm^-1^	θ_max_ = 28.3°, θ_min_ = 3.5°
ω–scan	*h* = −18→18
Absorption correction: multi-scan (*SADABS*; Krause *et al.*, 2015)	*k* = −6→6
*T*_min_ = 0.684, *T*_max_ = 0.746	*l* = −23→22
16322 measured reflections	

### 2-(Pyridin-2-yl)-1H-perimidine (1) Refinement

**Table:**

Refinement on *F*^2^	Primary atom site location: dual
Least-squares matrix: full	Hydrogen site location: difference Fourier map
*R*[*F*^2^ > 2σ(*F*^2^)] = 0.057	All H-atom parameters refined
*wR*(*F*^2^) = 0.140	*w* = 1/[σ^2^(*F*_o_^2^) + (0.057*P*)^2^ + 1.1159*P*] where *P* = (*F*_o_^2^ + 2*F*_c_^2^)/3
*S* = 1.05	(Δ/σ)_max_ < 0.001
2866 reflections	Δρ_max_ = 0.39 e Å^−^^3^
216 parameters	Δρ_min_ = −0.33 e Å^−^^3^
0 restraints	

### 2-(Pyridin-2-yl)-1H-perimidine (1) Special details

**Table:**

Geometry. All esds (except the esd in the dihedral angle between two l.s. planes) are estimated using the full covariance matrix. The cell esds are taken into account individually in the estimation of esds in distances, angles and torsion angles; correlations between esds in cell parameters are only used when they are defined by crystal symmetry. An approximate (isotropic) treatment of cell esds is used for estimating esds involving l.s. planes.

### 2-(Pyridin-2-yl)-1H-perimidine (1) Fractional atomic coordinates and isotropic or equivalent isotropic displacement parameters (Å^2^)

**Table:**

	*x*	*y*	*z*	*U*_iso_*/*U*_eq_	
N1	0.67572 (10)	0.4402 (3)	0.27627 (8)	0.0154 (3)	
N3	0.71391 (10)	0.0786 (3)	0.17704 (8)	0.0168 (3)	
N2	0.84493 (10)	0.5837 (3)	0.30290 (8)	0.0155 (3)	
C12	0.79419 (12)	0.2223 (3)	0.21158 (9)	0.0143 (3)	
C10	0.64357 (12)	0.6204 (3)	0.32655 (9)	0.0149 (3)	
C1	0.77359 (12)	0.4307 (3)	0.26757 (9)	0.0142 (3)	
C2	0.81956 (12)	0.7734 (3)	0.35506 (9)	0.0150 (3)	
C13	0.89068 (12)	0.1825 (3)	0.19733 (9)	0.0156 (3)	
C3	0.89194 (13)	0.9430 (3)	0.39534 (9)	0.0174 (3)	
C8	0.52046 (13)	0.8239 (4)	0.38981 (10)	0.0200 (4)	
C16	0.72925 (13)	−0.1115 (3)	0.12658 (10)	0.0189 (4)	
C15	0.82269 (13)	−0.1659 (3)	0.10900 (9)	0.0191 (4)	
C9	0.54570 (12)	0.6318 (3)	0.33781 (9)	0.0182 (3)	
C6	0.69204 (12)	0.9893 (3)	0.41877 (9)	0.0168 (3)	
C5	0.76845 (13)	1.1625 (3)	0.45756 (9)	0.0186 (3)	
C14	0.90460 (13)	−0.0168 (3)	0.14534 (10)	0.0181 (3)	
C11	0.71827 (12)	0.7951 (3)	0.36697 (9)	0.0145 (3)	
C7	0.59039 (13)	0.9992 (4)	0.42875 (10)	0.0201 (4)	
C4	0.86549 (13)	1.1376 (3)	0.44599 (9)	0.0187 (4)	
H3	0.9603 (15)	0.931 (4)	0.3862 (11)	0.020 (5)*	
H13	0.9452 (16)	0.282 (4)	0.2226 (12)	0.024 (5)*	
H8	0.4528 (16)	0.829 (4)	0.3965 (12)	0.024 (5)*	
H14	0.9698 (17)	−0.050 (5)	0.1362 (13)	0.031 (6)*	
H9	0.4957 (14)	0.510 (4)	0.3102 (11)	0.016 (5)*	
H7	0.5720 (16)	1.125 (5)	0.4625 (13)	0.030 (6)*	
H4	0.9168 (15)	1.252 (4)	0.4733 (12)	0.023 (5)*	
H16	0.6709 (15)	−0.203 (4)	0.1022 (12)	0.024 (5)*	
H1	0.6349 (17)	0.326 (5)	0.2490 (13)	0.032 (6)*	
H5	0.7526 (14)	1.292 (4)	0.4910 (12)	0.019 (5)*	
H15	0.8299 (15)	−0.302 (4)	0.0730 (12)	0.024 (5)*	

### 2-(Pyridin-2-yl)-1H-perimidine (1) Atomic displacement parameters (Å^2^)

**Table:**

	*U*^11^	*U*^22^	*U*^33^	*U*^12^	*U*^13^	*U*^23^
N1	0.0149 (6)	0.0159 (7)	0.0152 (6)	−0.0011 (5)	0.0022 (5)	−0.0026 (5)
N3	0.0164 (6)	0.0168 (7)	0.0166 (7)	−0.0005 (5)	0.0016 (5)	0.0003 (5)
N2	0.0172 (6)	0.0147 (7)	0.0147 (6)	0.0004 (5)	0.0033 (5)	0.0008 (5)
C12	0.0176 (7)	0.0129 (7)	0.0120 (7)	0.0013 (6)	0.0018 (6)	0.0024 (6)
C10	0.0172 (7)	0.0141 (7)	0.0128 (7)	0.0016 (6)	0.0016 (6)	0.0016 (6)
C1	0.0169 (7)	0.0127 (7)	0.0130 (7)	0.0016 (6)	0.0030 (6)	0.0023 (6)
C2	0.0183 (8)	0.0132 (7)	0.0135 (7)	0.0008 (6)	0.0034 (6)	0.0034 (6)
C13	0.0154 (7)	0.0155 (8)	0.0150 (7)	−0.0010 (6)	0.0011 (6)	−0.0002 (6)
C3	0.0194 (8)	0.0166 (8)	0.0158 (7)	−0.0012 (6)	0.0022 (6)	0.0024 (6)
C8	0.0166 (8)	0.0262 (9)	0.0177 (8)	0.0044 (7)	0.0047 (6)	0.0012 (7)
C16	0.0223 (8)	0.0160 (8)	0.0165 (8)	−0.0024 (7)	−0.0010 (6)	−0.0006 (6)
C15	0.0284 (9)	0.0142 (8)	0.0143 (7)	0.0027 (6)	0.0031 (6)	−0.0008 (6)
C9	0.0177 (8)	0.0197 (8)	0.0165 (8)	0.0013 (6)	0.0017 (6)	−0.0005 (6)
C6	0.0240 (8)	0.0141 (7)	0.0118 (7)	0.0020 (6)	0.0026 (6)	0.0025 (6)
C5	0.0288 (9)	0.0132 (8)	0.0135 (7)	0.0013 (7)	0.0035 (6)	−0.0006 (6)
C14	0.0181 (8)	0.0200 (8)	0.0167 (8)	0.0042 (6)	0.0044 (6)	0.0019 (6)
C11	0.0186 (8)	0.0129 (7)	0.0121 (7)	0.0014 (6)	0.0030 (6)	0.0026 (6)
C7	0.0248 (9)	0.0202 (8)	0.0162 (8)	0.0063 (7)	0.0059 (6)	−0.0017 (7)
C4	0.0260 (8)	0.0148 (8)	0.0137 (7)	−0.0047 (7)	−0.0002 (6)	0.0009 (6)

### 2-(Pyridin-2-yl)-1H-perimidine (1) Geometric parameters (Å, º)

**Table:**

N1—C10	1.387 (2)	C8—C9	1.410 (2)
N1—C1	1.365 (2)	C8—C7	1.371 (2)
N1—H1	0.87 (2)	C8—H8	0.95 (2)
N3—C12	1.344 (2)	C16—C15	1.387 (2)
N3—C16	1.341 (2)	C16—H16	0.94 (2)
N2—C1	1.292 (2)	C15—C14	1.384 (2)
N2—C2	1.404 (2)	C15—H15	0.94 (2)
C12—C1	1.493 (2)	C9—H9	0.97 (2)
C12—C13	1.392 (2)	C6—C5	1.417 (2)
C10—C9	1.379 (2)	C6—C11	1.420 (2)
C10—C11	1.417 (2)	C6—C7	1.422 (2)
C2—C3	1.381 (2)	C5—C4	1.374 (2)
C2—C11	1.432 (2)	C5—H5	0.93 (2)
C13—C14	1.387 (2)	C14—H14	0.94 (2)
C13—H13	0.93 (2)	C7—H7	0.93 (2)
C3—C4	1.409 (2)	C4—H4	0.95 (2)
C3—H3	0.97 (2)		
			
C10—N1—H1	122.0 (15)	N3—C16—H16	114.7 (13)
C1—N1—C10	121.71 (14)	C15—C16—H16	121.8 (13)
C1—N1—H1	116.3 (15)	C16—C15—H15	120.4 (12)
C16—N3—C12	117.31 (14)	C14—C15—C16	118.52 (15)
C1—N2—C2	117.11 (14)	C14—C15—H15	121.0 (12)
N3—C12—C1	115.40 (14)	C10—C9—C8	118.76 (15)
N3—C12—C13	123.28 (15)	C10—C9—H9	120.1 (11)
C13—C12—C1	121.31 (14)	C8—C9—H9	121.1 (11)
N1—C10—C11	115.76 (14)	C5—C6—C11	118.24 (15)
C9—C10—N1	123.16 (15)	C5—C6—C7	123.66 (15)
C9—C10—C11	121.09 (15)	C11—C6—C7	118.11 (15)
N1—C1—C12	114.06 (14)	C6—C5—H5	119.6 (12)
N2—C1—N1	125.35 (15)	C4—C5—C6	120.29 (15)
N2—C1—C12	120.59 (14)	C4—C5—H5	120.2 (12)
N2—C2—C11	120.58 (14)	C13—C14—H14	119.4 (14)
C3—C2—N2	120.44 (14)	C15—C14—C13	119.24 (15)
C3—C2—C11	118.98 (15)	C15—C14—H14	121.4 (14)
C12—C13—H13	121.8 (13)	C10—C11—C2	119.48 (14)
C14—C13—C12	118.23 (15)	C10—C11—C6	119.78 (14)
C14—C13—H13	119.9 (13)	C6—C11—C2	120.74 (15)
C2—C3—C4	120.17 (15)	C8—C7—C6	120.58 (15)
C2—C3—H3	118.9 (12)	C8—C7—H7	120.3 (13)
C4—C3—H3	120.9 (12)	C6—C7—H7	119.2 (13)
C9—C8—H8	117.3 (13)	C3—C4—H4	118.7 (12)
C7—C8—C9	121.68 (15)	C5—C4—C3	121.57 (15)
C7—C8—H8	121.0 (13)	C5—C4—H4	119.8 (12)
N3—C16—C15	123.42 (15)		
			
N1—C10—C9—C8	−179.50 (15)	C13—C12—C1—N1	−178.61 (14)
N1—C10—C11—C2	−1.0 (2)	C13—C12—C1—N2	1.6 (2)
N1—C10—C11—C6	179.00 (14)	C3—C2—C11—C10	−179.38 (14)
N3—C12—C1—N1	1.1 (2)	C3—C2—C11—C6	0.6 (2)
N3—C12—C1—N2	−178.67 (14)	C16—N3—C12—C1	−179.71 (13)
N3—C12—C13—C14	−0.5 (2)	C16—N3—C12—C13	0.0 (2)
N3—C16—C15—C14	0.1 (3)	C16—C15—C14—C13	−0.6 (2)
N2—C2—C3—C4	178.31 (14)	C9—C10—C11—C2	178.63 (15)
N2—C2—C11—C10	1.0 (2)	C9—C10—C11—C6	−1.3 (2)
N2—C2—C11—C6	−179.07 (14)	C9—C8—C7—C6	−1.1 (3)
C12—N3—C16—C15	0.2 (2)	C6—C5—C4—C3	0.5 (2)
C12—C13—C14—C15	0.8 (2)	C5—C6—C11—C10	−179.34 (14)
C10—N1—C1—N2	−0.8 (2)	C5—C6—C11—C2	0.7 (2)
C10—N1—C1—C12	179.41 (13)	C5—C6—C7—C8	−179.48 (16)
C1—N1—C10—C9	−178.72 (15)	C11—C10—C9—C8	0.8 (2)
C1—N1—C10—C11	1.0 (2)	C11—C2—C3—C4	−1.3 (2)
C1—N2—C2—C3	179.62 (14)	C11—C6—C5—C4	−1.3 (2)
C1—N2—C2—C11	−0.7 (2)	C11—C6—C7—C8	0.6 (2)
C1—C12—C13—C14	179.19 (14)	C7—C8—C9—C10	0.4 (3)
C2—N2—C1—N1	0.7 (2)	C7—C6—C5—C4	178.83 (16)
C2—N2—C1—C12	−179.58 (13)	C7—C6—C11—C10	0.6 (2)
C2—C3—C4—C5	0.8 (2)	C7—C6—C11—C2	−179.37 (14)

### 1-Methyl-2-(pyridin-2-yl)-1H-perimidine (2) Crystal data

**Table:**

C_17_H_13_N_3_	*F*(000) = 544
*M**_r_* = 259.30	*D*_x_ = 1.397 Mg m^−^^3^
Monoclinic, *P*2_1_/*c*	Mo *K*α radiation, λ = 0.71073 Å
*a* = 7.5095 (2) Å	Cell parameters from 5485 reflections
*b* = 12.1216 (3) Å	θ = 2.3–30.5°
*c* = 13.5616 (4) Å	µ = 0.09 mm^−^^1^
β = 92.547 (1)°	*T* = 100 K
*V* = 1233.25 (6) Å^3^	Block, orange
*Z* = 4	0.34 × 0.12 × 0.11 mm

### 1-Methyl-2-(pyridin-2-yl)-1H-perimidine (2) Data collection

**Table:**

Bruker D8 Venture diffractometer	3280 independent reflections
Radiation source: microfocus sealed X-ray tube, Incoatec IµS microsource	2803 reflections with *I* > 2σ(*I*)
Focusing mirrors monochromator	*R*_int_ = 0.035
Detector resolution: 10.4 pixels mm^-1^	θ_max_ = 29.0°, θ_min_ = 2.3°
ω–scan	*h* = −10→10
Absorption correction: multi-scan (*SADABS*; Krause *et al.*, 2015)	*k* = −15→16
*T*_min_ = 0.685, *T*_max_ = 0.746	*l* = −18→18
14029 measured reflections	

### 1-Methyl-2-(pyridin-2-yl)-1H-perimidine (2) Refinement

**Table:**

Refinement on *F*^2^	0 restraints
Least-squares matrix: full	Hydrogen site location: difference Fourier map
*R*[*F*^2^ > 2σ(*F*^2^)] = 0.060	All H-atom parameters refined
*wR*(*F*^2^) = 0.146	*w* = 1/[σ^2^(*F*_o_^2^) + (0.0706*P*)^2^ + 0.7754*P*] where *P* = (*F*_o_^2^ + 2*F*_c_^2^)/3
*S* = 1.04	(Δ/σ)_max_ < 0.001
3280 reflections	Δρ_max_ = 0.45 e Å^−^^3^
233 parameters	Δρ_min_ = −0.33 e Å^−^^3^

### 1-Methyl-2-(pyridin-2-yl)-1H-perimidine (2) Special details

**Table:**

Geometry. All esds (except the esd in the dihedral angle between two l.s. planes) are estimated using the full covariance matrix. The cell esds are taken into account individually in the estimation of esds in distances, angles and torsion angles; correlations between esds in cell parameters are only used when they are defined by crystal symmetry. An approximate (isotropic) treatment of cell esds is used for estimating esds involving l.s. planes.

### 1-Methyl-2-(pyridin-2-yl)-1H-perimidine (2) Fractional atomic coordinates and isotropic or equivalent isotropic displacement parameters (Å^2^)

**Table:**

	*x*	*y*	*z*	*U*_iso_*/*U*_eq_	
N2	0.76302 (17)	0.66381 (10)	0.66410 (9)	0.0173 (3)	
N3	0.79159 (17)	0.88961 (10)	0.52437 (10)	0.0197 (3)	
N1	0.69884 (16)	0.65068 (10)	0.49124 (9)	0.0153 (3)	
C11	0.79946 (19)	0.48774 (12)	0.57893 (11)	0.0160 (3)	
C1	0.71526 (19)	0.70642 (12)	0.57923 (11)	0.0155 (3)	
C13	0.68606 (19)	0.82886 (12)	0.58029 (11)	0.0157 (3)	
C2	0.80957 (19)	0.55209 (12)	0.66662 (11)	0.0166 (3)	
C14	0.5640 (2)	0.87417 (13)	0.64331 (11)	0.0177 (3)	
C6	0.85120 (19)	0.37474 (12)	0.58061 (12)	0.0187 (3)	
C12	0.6294 (2)	0.70168 (13)	0.39951 (11)	0.0194 (3)	
C3	0.8700 (2)	0.50420 (13)	0.75469 (12)	0.0208 (3)	
C16	0.6670 (2)	1.05215 (13)	0.59611 (12)	0.0206 (3)	
C15	0.5551 (2)	0.98867 (13)	0.65099 (12)	0.0200 (3)	
C17	0.7802 (2)	0.99943 (13)	0.53330 (13)	0.0219 (3)	
C9	0.7297 (2)	0.47699 (13)	0.40235 (12)	0.0202 (3)	
C10	0.74095 (19)	0.53778 (12)	0.48835 (11)	0.0156 (3)	
C5	0.9117 (2)	0.32879 (13)	0.67236 (13)	0.0218 (3)	
C7	0.8416 (2)	0.31473 (12)	0.49048 (13)	0.0220 (3)	
C4	0.9205 (2)	0.39232 (13)	0.75648 (13)	0.0226 (3)	
C8	0.7817 (2)	0.36497 (13)	0.40491 (12)	0.0228 (3)	
H12A	0.603 (3)	0.7776 (18)	0.4103 (15)	0.029 (5)*	
H3	0.879 (3)	0.5482 (16)	0.8163 (15)	0.027 (5)*	
H5	0.951 (3)	0.2505 (18)	0.6727 (15)	0.030 (5)*	
H7	0.882 (3)	0.2398 (17)	0.4889 (14)	0.025 (5)*	
H16	0.663 (3)	1.1313 (17)	0.5991 (14)	0.026 (5)*	
H12B	0.723 (3)	0.7008 (16)	0.3497 (14)	0.023 (5)*	
H4	0.961 (3)	0.3617 (18)	0.8184 (16)	0.033 (5)*	
H17	0.856 (3)	1.0433 (17)	0.4948 (15)	0.028 (5)*	
H9	0.695 (3)	0.5092 (17)	0.3402 (15)	0.026 (5)*	
H12C	0.518 (3)	0.6600 (16)	0.3771 (14)	0.025 (5)*	
H15	0.474 (3)	1.0204 (17)	0.6955 (15)	0.027 (5)*	
H14	0.489 (3)	0.8271 (16)	0.6786 (14)	0.023 (5)*	
H8	0.777 (3)	0.3237 (17)	0.3434 (16)	0.033 (5)*	

### 1-Methyl-2-(pyridin-2-yl)-1H-perimidine (2) Atomic displacement parameters (Å^2^)

**Table:**

	*U*^11^	*U*^22^	*U*^33^	*U*^12^	*U*^13^	*U*^23^
N2	0.0187 (6)	0.0155 (6)	0.0178 (6)	0.0007 (5)	0.0024 (5)	0.0012 (5)
N3	0.0193 (6)	0.0155 (6)	0.0248 (7)	0.0029 (5)	0.0045 (5)	0.0026 (5)
N1	0.0172 (6)	0.0128 (6)	0.0160 (6)	0.0024 (5)	0.0005 (5)	0.0006 (4)
C11	0.0123 (6)	0.0151 (7)	0.0208 (7)	−0.0008 (5)	0.0030 (5)	0.0023 (5)
C1	0.0130 (6)	0.0144 (6)	0.0192 (7)	0.0011 (5)	0.0023 (5)	−0.0003 (5)
C13	0.0146 (6)	0.0148 (6)	0.0174 (7)	0.0021 (5)	−0.0011 (5)	−0.0006 (5)
C2	0.0142 (6)	0.0158 (7)	0.0200 (7)	0.0003 (5)	0.0030 (5)	0.0025 (5)
C14	0.0162 (7)	0.0194 (7)	0.0175 (7)	0.0007 (6)	0.0009 (5)	−0.0003 (6)
C6	0.0143 (6)	0.0147 (7)	0.0274 (8)	−0.0006 (5)	0.0045 (6)	0.0027 (6)
C12	0.0207 (7)	0.0182 (7)	0.0189 (7)	0.0038 (6)	−0.0024 (6)	0.0013 (6)
C3	0.0200 (7)	0.0217 (8)	0.0206 (7)	−0.0001 (6)	0.0017 (6)	0.0040 (6)
C16	0.0174 (7)	0.0143 (7)	0.0299 (8)	0.0027 (6)	−0.0020 (6)	−0.0023 (6)
C15	0.0184 (7)	0.0216 (7)	0.0199 (7)	0.0046 (6)	−0.0006 (6)	−0.0045 (6)
C17	0.0188 (7)	0.0167 (7)	0.0304 (8)	0.0010 (6)	0.0030 (6)	0.0035 (6)
C9	0.0205 (7)	0.0184 (7)	0.0219 (7)	−0.0017 (6)	0.0026 (6)	−0.0019 (6)
C10	0.0135 (6)	0.0131 (6)	0.0205 (7)	−0.0004 (5)	0.0033 (5)	−0.0004 (5)
C5	0.0181 (7)	0.0144 (7)	0.0329 (8)	0.0011 (6)	0.0035 (6)	0.0076 (6)
C7	0.0217 (7)	0.0118 (7)	0.0330 (8)	−0.0003 (6)	0.0076 (6)	−0.0015 (6)
C4	0.0191 (7)	0.0223 (8)	0.0262 (8)	−0.0001 (6)	0.0010 (6)	0.0097 (6)
C8	0.0234 (8)	0.0190 (7)	0.0265 (8)	−0.0037 (6)	0.0066 (6)	−0.0070 (6)

### 1-Methyl-2-(pyridin-2-yl)-1H-perimidine (2) Geometric parameters (Å, º)

**Table:**

N2—C1	1.2974 (19)	C12—H12B	0.996 (19)
N2—C2	1.3986 (18)	C12—H12C	1.01 (2)
N3—C13	1.341 (2)	C3—C4	1.408 (2)
N3—C17	1.340 (2)	C3—H3	0.99 (2)
N1—C1	1.3720 (18)	C16—C15	1.381 (2)
N1—C12	1.4644 (18)	C16—C17	1.386 (2)
N1—C10	1.4055 (18)	C16—H16	0.96 (2)
C11—C2	1.421 (2)	C15—H15	0.96 (2)
C11—C6	1.424 (2)	C17—H17	0.95 (2)
C11—C10	1.422 (2)	C9—C10	1.379 (2)
C1—C13	1.5005 (19)	C9—C8	1.413 (2)
C13—C14	1.393 (2)	C9—H9	0.95 (2)
C2—C3	1.386 (2)	C5—C4	1.376 (2)
C14—C15	1.394 (2)	C5—H5	0.99 (2)
C14—H14	0.94 (2)	C7—C8	1.369 (2)
C6—C5	1.419 (2)	C7—H7	0.96 (2)
C6—C7	1.421 (2)	C4—H4	0.96 (2)
C12—H12A	0.95 (2)	C8—H8	0.97 (2)
			
C1—N2—C2	117.78 (13)	C2—C3—H3	120.6 (12)
C17—N3—C13	116.91 (13)	C4—C3—H3	119.6 (12)
C1—N1—C12	123.08 (12)	C15—C16—C17	118.60 (14)
C1—N1—C10	119.48 (12)	C15—C16—H16	120.9 (12)
C10—N1—C12	117.35 (12)	C17—C16—H16	120.4 (12)
C2—C11—C6	120.68 (13)	C14—C15—H15	118.8 (12)
C2—C11—C10	119.45 (13)	C16—C15—C14	118.81 (14)
C10—C11—C6	119.86 (14)	C16—C15—H15	122.3 (12)
N2—C1—N1	125.88 (13)	N3—C17—C16	123.87 (15)
N2—C1—C13	114.84 (13)	N3—C17—H17	117.6 (12)
N1—C1—C13	119.20 (12)	C16—C17—H17	118.5 (12)
N3—C13—C1	116.58 (13)	C10—C9—C8	119.10 (15)
N3—C13—C14	123.48 (14)	C10—C9—H9	122.2 (12)
C14—C13—C1	119.74 (13)	C8—C9—H9	118.6 (12)
N2—C2—C11	120.45 (13)	N1—C10—C11	116.90 (13)
C3—C2—N2	119.91 (14)	C9—C10—N1	122.59 (13)
C3—C2—C11	119.62 (14)	C9—C10—C11	120.51 (14)
C13—C14—C15	118.27 (14)	C6—C5—H5	117.5 (12)
C13—C14—H14	119.6 (12)	C4—C5—C6	120.59 (14)
C15—C14—H14	122.2 (12)	C4—C5—H5	121.9 (12)
C5—C6—C11	117.91 (14)	C6—C7—H7	120.0 (12)
C5—C6—C7	123.71 (14)	C8—C7—C6	120.24 (14)
C7—C6—C11	118.37 (14)	C8—C7—H7	119.7 (12)
N1—C12—H12A	110.2 (12)	C3—C4—H4	117.7 (13)
N1—C12—H12B	109.7 (11)	C5—C4—C3	121.47 (15)
N1—C12—H12C	107.6 (11)	C5—C4—H4	120.9 (13)
H12A—C12—H12B	105.7 (17)	C9—C8—H8	118.3 (12)
H12A—C12—H12C	110.7 (17)	C7—C8—C9	121.90 (14)
H12B—C12—H12C	113.0 (15)	C7—C8—H8	119.8 (12)
C2—C3—C4	119.73 (15)		
			
N2—C1—C13—N3	118.34 (15)	C6—C11—C2—C3	−0.2 (2)
N2—C1—C13—C14	−56.73 (19)	C6—C11—C10—N1	−177.46 (12)
N2—C2—C3—C4	−178.61 (13)	C6—C11—C10—C9	1.6 (2)
N3—C13—C14—C15	−2.1 (2)	C6—C5—C4—C3	0.1 (2)
N1—C1—C13—N3	−58.57 (18)	C6—C7—C8—C9	0.7 (2)
N1—C1—C13—C14	126.37 (15)	C12—N1—C1—N2	174.50 (14)
C11—C2—C3—C4	−0.1 (2)	C12—N1—C1—C13	−9.0 (2)
C11—C6—C5—C4	−0.3 (2)	C12—N1—C10—C11	−176.81 (13)
C11—C6—C7—C8	−0.8 (2)	C12—N1—C10—C9	4.2 (2)
C1—N2—C2—C11	−1.7 (2)	C15—C16—C17—N3	−2.0 (2)
C1—N2—C2—C3	176.77 (14)	C17—N3—C13—C1	−172.77 (13)
C1—N1—C10—C11	−0.12 (19)	C17—N3—C13—C14	2.1 (2)
C1—N1—C10—C9	−179.15 (14)	C17—C16—C15—C14	1.9 (2)
C1—C13—C14—C15	172.63 (13)	C10—N1—C1—N2	−2.0 (2)
C13—N3—C17—C16	0.0 (2)	C10—N1—C1—C13	174.54 (12)
C13—C14—C15—C16	0.0 (2)	C10—C11—C2—N2	−0.2 (2)
C2—N2—C1—N1	2.9 (2)	C10—C11—C2—C3	−178.68 (13)
C2—N2—C1—C13	−173.79 (12)	C10—C11—C6—C5	178.90 (13)
C2—C11—C6—C5	0.4 (2)	C10—C11—C6—C7	−0.3 (2)
C2—C11—C6—C7	−178.86 (13)	C10—C9—C8—C7	0.6 (2)
C2—C11—C10—N1	1.1 (2)	C5—C6—C7—C8	−179.97 (15)
C2—C11—C10—C9	−179.87 (13)	C7—C6—C5—C4	178.87 (15)
C2—C3—C4—C5	0.2 (2)	C8—C9—C10—N1	177.31 (13)
C6—C11—C2—N2	178.32 (13)	C8—C9—C10—C11	−1.7 (2)

### 1,3-Dimethyl-2-(pyridin-2-yl)-1H-perimidinium iodide (3) Crystal data

**Table:**

C_18_H_16_N_3_^+^·I^−^	*F*(000) = 792
*M**_r_* = 401.24	*D*_x_ = 1.589 Mg m^−^^3^
Monoclinic, *P*2_1_/*n*	Mo *K*α radiation, λ = 0.71073 Å
*a* = 9.8821 (2) Å	Cell parameters from 9859 reflections
*b* = 9.7125 (2) Å	θ = 2.3–28.3°
*c* = 17.9839 (4) Å	µ = 1.91 mm^−^^1^
β = 103.676 (1)°	*T* = 230 K
*V* = 1677.15 (6) Å^3^	Block, yellow
*Z* = 4	0.32 × 0.18 × 0.13 mm

### 1,3-Dimethyl-2-(pyridin-2-yl)-1H-perimidinium iodide (3) Data collection

**Table:**

Bruker SMART APEXII diffractometer	4146 independent reflections
Radiation source: fine-focus sealed X-ray tube, X-ray tube	3700 reflections with *I* > 2σ(*I*)
Mirror optics monochromator	*R*_int_ = 0.026
Detector resolution: 7.9 pixels mm^-1^	θ_max_ = 28.4°, θ_min_ = 2.3°
ω scan	*h* = −13→13
Absorption correction: multi-scan (*SADABS*; Krause *et al.*, 2015)	*k* = −12→12
*T*_min_ = 0.668, *T*_max_ = 0.746	*l* = −23→23
28389 measured reflections	

### 1,3-Dimethyl-2-(pyridin-2-yl)-1H-perimidinium iodide (3) Refinement

**Table:**

Refinement on *F*^2^	Primary atom site location: dual
Least-squares matrix: full	Hydrogen site location: inferred from neighbouring sites
*R*[*F*^2^ > 2σ(*F*^2^)] = 0.028	H-atom parameters constrained
*wR*(*F*^2^) = 0.067	*w* = 1/[σ^2^(*F*_o_^2^) + (0.0273*P*)^2^ + 1.1509*P*] where *P* = (*F*_o_^2^ + 2*F*_c_^2^)/3
*S* = 1.06	(Δ/σ)_max_ < 0.001
4146 reflections	Δρ_max_ = 0.72 e Å^−^^3^
201 parameters	Δρ_min_ = −0.46 e Å^−^^3^
0 restraints	

### 1,3-Dimethyl-2-(pyridin-2-yl)-1H-perimidinium iodide (3) Special details

**Table:**

Geometry. All esds (except the esd in the dihedral angle between two l.s. planes) are estimated using the full covariance matrix. The cell esds are taken into account individually in the estimation of esds in distances, angles and torsion angles; correlations between esds in cell parameters are only used when they are defined by crystal symmetry. An approximate (isotropic) treatment of cell esds is used for estimating esds involving l.s. planes.

### 1,3-Dimethyl-2-(pyridin-2-yl)-1H-perimidinium iodide (3) Fractional atomic coordinates and isotropic or equivalent isotropic displacement parameters (Å^2^)

**Table:**

	*x*	*y*	*z*	*U*_iso_*/*U*_eq_	
I1	0.46311 (2)	0.43924 (2)	0.27610 (2)	0.05057 (7)	
N2	0.70014 (19)	0.6145 (2)	0.48068 (10)	0.0417 (4)	
C14	0.6404 (2)	0.8060 (2)	0.39271 (12)	0.0389 (4)	
N1	0.78491 (17)	0.61380 (19)	0.37060 (10)	0.0380 (4)	
C11	0.8424 (2)	0.4240 (2)	0.45912 (13)	0.0397 (4)	
C15	0.5033 (2)	0.8101 (2)	0.35210 (14)	0.0475 (5)	
H15	0.453130	0.728579	0.336938	0.057*	
C1	0.7119 (2)	0.6712 (2)	0.41527 (12)	0.0377 (4)	
N3	0.7182 (2)	0.9164 (2)	0.41510 (15)	0.0633 (6)	
C2	0.7651 (2)	0.4869 (2)	0.50629 (13)	0.0425 (5)	
C10	0.8527 (2)	0.4851 (2)	0.38939 (12)	0.0401 (4)	
C3	0.7545 (3)	0.4271 (3)	0.57385 (16)	0.0573 (6)	
H3	0.701812	0.469174	0.604759	0.069*	
C6	0.9113 (2)	0.2971 (2)	0.48227 (15)	0.0494 (5)	
C16	0.4421 (3)	0.9371 (3)	0.33438 (17)	0.0562 (6)	
H16	0.348626	0.943924	0.307067	0.067*	
C7	0.9857 (3)	0.2348 (3)	0.43274 (18)	0.0619 (7)	
H7	1.031867	0.150768	0.446700	0.074*	
C13	0.6248 (3)	0.6850 (3)	0.53130 (15)	0.0618 (7)	
H13A	0.589318	0.772451	0.508734	0.093*	
H13B	0.687651	0.701041	0.580789	0.093*	
H13C	0.547719	0.628024	0.537689	0.093*	
C8	0.9909 (3)	0.2948 (3)	0.36572 (18)	0.0646 (7)	
H8	1.039315	0.250527	0.333392	0.078*	
C17	0.5194 (3)	1.0531 (2)	0.35717 (18)	0.0600 (7)	
H17	0.480165	1.141064	0.346141	0.072*	
C12	0.7915 (3)	0.6767 (3)	0.29709 (13)	0.0525 (6)	
H12A	0.741265	0.619574	0.255397	0.079*	
H12B	0.887977	0.684564	0.294207	0.079*	
H12C	0.749586	0.767565	0.293224	0.079*	
C18	0.6552 (4)	1.0379 (3)	0.3964 (2)	0.0725 (9)	
H18	0.707770	1.118127	0.411245	0.087*	
C9	0.9255 (3)	0.4220 (3)	0.34300 (16)	0.0536 (6)	
H9	0.931871	0.462969	0.296594	0.064*	
C5	0.8993 (3)	0.2393 (3)	0.55276 (18)	0.0622 (7)	
H5	0.944762	0.155928	0.569505	0.075*	
C4	0.8238 (3)	0.3020 (3)	0.59604 (17)	0.0659 (8)	
H4	0.817190	0.261112	0.642411	0.079*	

### 1,3-Dimethyl-2-(pyridin-2-yl)-1H-perimidinium iodide (3) Atomic displacement parameters (Å^2^)

**Table:**

	*U*^11^	*U*^22^	*U*^33^	*U*^12^	*U*^13^	*U*^23^
I1	0.05271 (10)	0.04098 (9)	0.05737 (11)	−0.00419 (6)	0.01171 (7)	−0.01150 (6)
N2	0.0404 (9)	0.0444 (10)	0.0404 (9)	0.0032 (8)	0.0100 (7)	0.0044 (8)
C14	0.0395 (10)	0.0339 (10)	0.0430 (10)	−0.0019 (8)	0.0090 (8)	0.0019 (8)
N1	0.0339 (8)	0.0395 (9)	0.0389 (9)	−0.0004 (7)	0.0054 (7)	0.0043 (7)
C11	0.0312 (9)	0.0350 (10)	0.0467 (11)	−0.0059 (8)	−0.0029 (8)	0.0020 (8)
C15	0.0396 (11)	0.0363 (11)	0.0629 (14)	−0.0025 (9)	0.0049 (10)	0.0001 (10)
C1	0.0313 (9)	0.0375 (10)	0.0416 (10)	−0.0021 (8)	0.0035 (8)	0.0033 (8)
N3	0.0528 (12)	0.0445 (12)	0.0820 (16)	−0.0107 (10)	−0.0054 (11)	−0.0028 (11)
C2	0.0363 (10)	0.0416 (11)	0.0463 (11)	−0.0045 (9)	0.0031 (9)	0.0085 (9)
C10	0.0326 (10)	0.0401 (11)	0.0427 (11)	−0.0011 (8)	−0.0005 (8)	−0.0030 (9)
C3	0.0531 (14)	0.0652 (17)	0.0532 (14)	−0.0032 (12)	0.0118 (11)	0.0165 (12)
C6	0.0384 (11)	0.0356 (11)	0.0644 (14)	−0.0056 (9)	−0.0072 (10)	0.0028 (10)
C16	0.0457 (13)	0.0501 (14)	0.0697 (16)	0.0107 (11)	0.0079 (12)	0.0046 (12)
C7	0.0487 (14)	0.0378 (12)	0.088 (2)	0.0056 (11)	−0.0068 (13)	−0.0064 (12)
C13	0.0679 (16)	0.0709 (17)	0.0523 (14)	0.0169 (14)	0.0255 (12)	0.0077 (13)
C8	0.0539 (15)	0.0597 (16)	0.0761 (18)	0.0122 (13)	0.0072 (13)	−0.0189 (14)
C17	0.0728 (18)	0.0353 (12)	0.0735 (17)	0.0086 (11)	0.0201 (14)	0.0032 (11)
C12	0.0540 (13)	0.0605 (15)	0.0452 (12)	0.0073 (12)	0.0163 (10)	0.0136 (11)
C18	0.075 (2)	0.0353 (13)	0.099 (2)	−0.0130 (13)	0.0047 (17)	−0.0059 (13)
C9	0.0497 (13)	0.0563 (14)	0.0530 (13)	0.0056 (11)	0.0086 (11)	−0.0080 (11)
C5	0.0538 (14)	0.0416 (13)	0.0792 (18)	−0.0041 (11)	−0.0085 (13)	0.0203 (12)
C4	0.0599 (16)	0.0647 (17)	0.0673 (17)	−0.0064 (14)	0.0036 (13)	0.0311 (14)

### 1,3-Dimethyl-2-(pyridin-2-yl)-1H-perimidinium iodide (3) Geometric parameters (Å, º)

**Table:**

N2—C1	1.328 (3)	C6—C5	1.417 (4)
N2—C2	1.422 (3)	C16—H16	0.9400
N2—C13	1.474 (3)	C16—C17	1.369 (4)
C14—C15	1.379 (3)	C7—H7	0.9400
C14—C1	1.497 (3)	C7—C8	1.351 (4)
C14—N3	1.326 (3)	C13—H13A	0.9700
N1—C1	1.323 (3)	C13—H13B	0.9700
N1—C10	1.421 (3)	C13—H13C	0.9700
N1—C12	1.472 (3)	C8—H8	0.9400
C11—C2	1.408 (3)	C8—C9	1.409 (4)
C11—C10	1.413 (3)	C17—H17	0.9400
C11—C6	1.422 (3)	C17—C18	1.368 (4)
C15—H15	0.9400	C12—H12A	0.9700
C15—C16	1.378 (3)	C12—H12B	0.9700
N3—C18	1.339 (4)	C12—H12C	0.9700
C2—C3	1.373 (3)	C18—H18	0.9400
C10—C9	1.369 (3)	C9—H9	0.9400
C3—H3	0.9400	C5—H5	0.9400
C3—C4	1.405 (4)	C5—C4	1.345 (4)
C6—C7	1.417 (4)	C4—H4	0.9400
			
C1—N2—C2	121.39 (19)	C6—C7—H7	119.6
C1—N2—C13	121.1 (2)	C8—C7—C6	120.8 (2)
C2—N2—C13	117.40 (19)	C8—C7—H7	119.6
C15—C14—C1	120.71 (19)	N2—C13—H13A	109.5
N3—C14—C15	124.3 (2)	N2—C13—H13B	109.5
N3—C14—C1	114.99 (19)	N2—C13—H13C	109.5
C1—N1—C10	121.36 (18)	H13A—C13—H13B	109.5
C1—N1—C12	121.10 (18)	H13A—C13—H13C	109.5
C10—N1—C12	117.43 (18)	H13B—C13—H13C	109.5
C2—C11—C10	121.08 (19)	C7—C8—H8	119.2
C2—C11—C6	119.4 (2)	C7—C8—C9	121.7 (3)
C10—C11—C6	119.6 (2)	C9—C8—H8	119.2
C14—C15—H15	120.9	C16—C17—H17	120.8
C16—C15—C14	118.2 (2)	C18—C17—C16	118.4 (2)
C16—C15—H15	120.9	C18—C17—H17	120.8
N2—C1—C14	117.88 (19)	N1—C12—H12A	109.5
N1—C1—N2	122.52 (19)	N1—C12—H12B	109.5
N1—C1—C14	119.60 (18)	N1—C12—H12C	109.5
C14—N3—C18	115.8 (2)	H12A—C12—H12B	109.5
C11—C2—N2	116.78 (19)	H12A—C12—H12C	109.5
C3—C2—N2	122.2 (2)	H12B—C12—H12C	109.5
C3—C2—C11	121.0 (2)	N3—C18—C17	124.5 (2)
C11—C10—N1	116.85 (19)	N3—C18—H18	117.8
C9—C10—N1	122.4 (2)	C17—C18—H18	117.8
C9—C10—C11	120.8 (2)	C10—C9—C8	119.1 (3)
C2—C3—H3	120.6	C10—C9—H9	120.4
C2—C3—C4	118.9 (3)	C8—C9—H9	120.4
C4—C3—H3	120.6	C6—C5—H5	119.6
C7—C6—C11	118.0 (2)	C4—C5—C6	120.9 (2)
C5—C6—C11	118.0 (2)	C4—C5—H5	119.6
C5—C6—C7	123.9 (2)	C3—C4—H4	119.1
C15—C16—H16	120.5	C5—C4—C3	121.8 (3)
C17—C16—C15	118.9 (2)	C5—C4—H4	119.1
C17—C16—H16	120.5		
			
N2—C2—C3—C4	−178.8 (2)	C2—C11—C6—C5	−0.2 (3)
C14—C15—C16—C17	0.4 (4)	C2—C3—C4—C5	−0.3 (4)
C14—N3—C18—C17	0.3 (5)	C10—N1—C1—N2	1.7 (3)
N1—C10—C9—C8	179.6 (2)	C10—N1—C1—C14	−179.10 (18)
C11—C2—C3—C4	0.7 (4)	C10—C11—C2—N2	−0.7 (3)
C11—C10—C9—C8	−0.2 (4)	C10—C11—C2—C3	179.7 (2)
C11—C6—C7—C8	0.2 (4)	C10—C11—C6—C7	−1.6 (3)
C11—C6—C5—C4	0.6 (4)	C10—C11—C6—C5	179.6 (2)
C15—C14—C1—N2	−87.1 (3)	C6—C11—C2—N2	179.11 (18)
C15—C14—C1—N1	93.6 (3)	C6—C11—C2—C3	−0.5 (3)
C15—C14—N3—C18	0.6 (4)	C6—C11—C10—N1	−178.17 (18)
C15—C16—C17—C18	0.4 (5)	C6—C11—C10—C9	1.6 (3)
C1—N2—C2—C11	0.2 (3)	C6—C7—C8—C9	1.2 (4)
C1—N2—C2—C3	179.7 (2)	C6—C5—C4—C3	−0.4 (4)
C1—C14—C15—C16	178.6 (2)	C16—C17—C18—N3	−0.8 (5)
C1—C14—N3—C18	−179.0 (3)	C7—C6—C5—C4	−178.1 (3)
C1—N1—C10—C11	−2.1 (3)	C7—C8—C9—C10	−1.3 (4)
C1—N1—C10—C9	178.1 (2)	C13—N2—C1—C14	−2.8 (3)
N3—C14—C15—C16	−1.0 (4)	C13—N2—C1—N1	176.4 (2)
N3—C14—C1—N2	92.5 (3)	C13—N2—C2—C11	−177.0 (2)
N3—C14—C1—N1	−86.8 (3)	C13—N2—C2—C3	2.6 (3)
C2—N2—C1—C14	−179.88 (18)	C12—N1—C1—N2	177.7 (2)
C2—N2—C1—N1	−0.6 (3)	C12—N1—C1—C14	−3.1 (3)
C2—C11—C10—N1	1.7 (3)	C12—N1—C10—C11	−178.33 (19)
C2—C11—C10—C9	−178.6 (2)	C12—N1—C10—C9	1.9 (3)
C2—C11—C6—C7	178.6 (2)	C5—C6—C7—C8	178.9 (3)
