# Supplementary figures and images for: Synthesis and comparative structural study of 2-(pyridin-2-yl)-1H-perimidine and its mono- and di-N-methyl­ated analogues
Source: Acta Crystallogr E Crystallogr Commun. 2021 Jan 8;77(Pt 2):96–100. doi: 10.1107/S205698902100013X (PMC7869552; doi:10.1107/S205698902100013X)

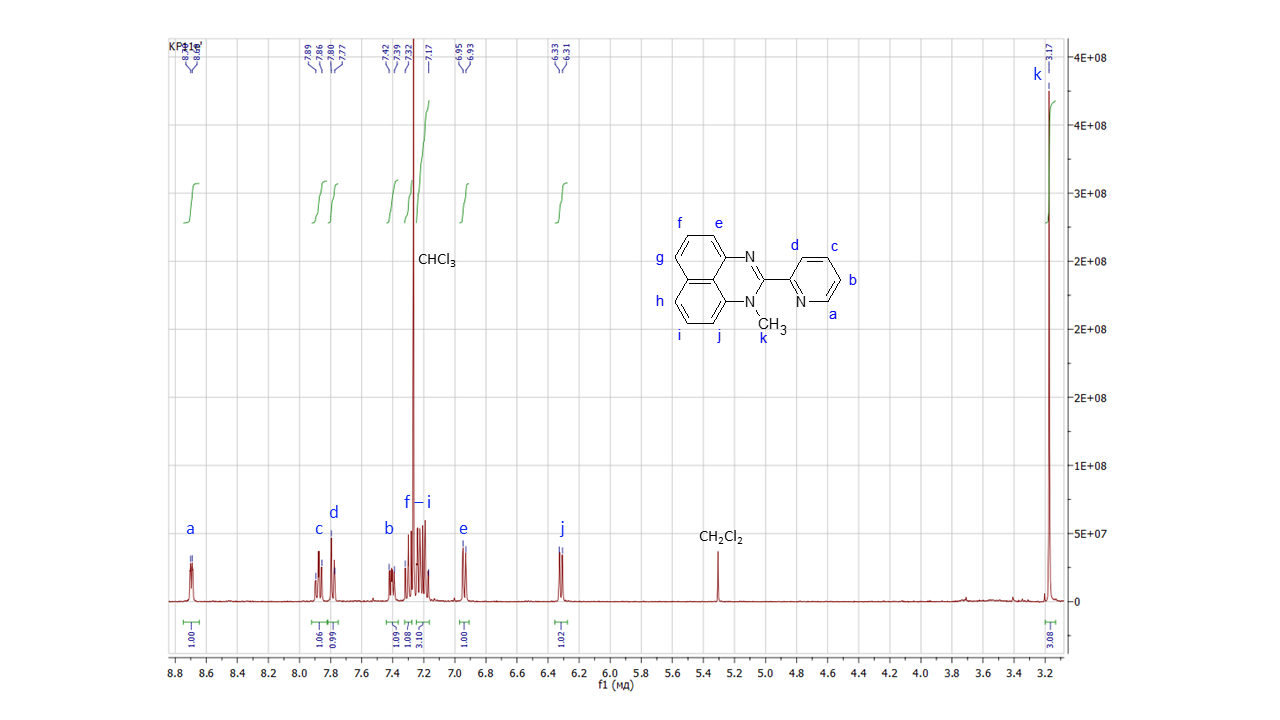

Supplement: Supplementary file 11 [file e-77-00096-sup11.tif]

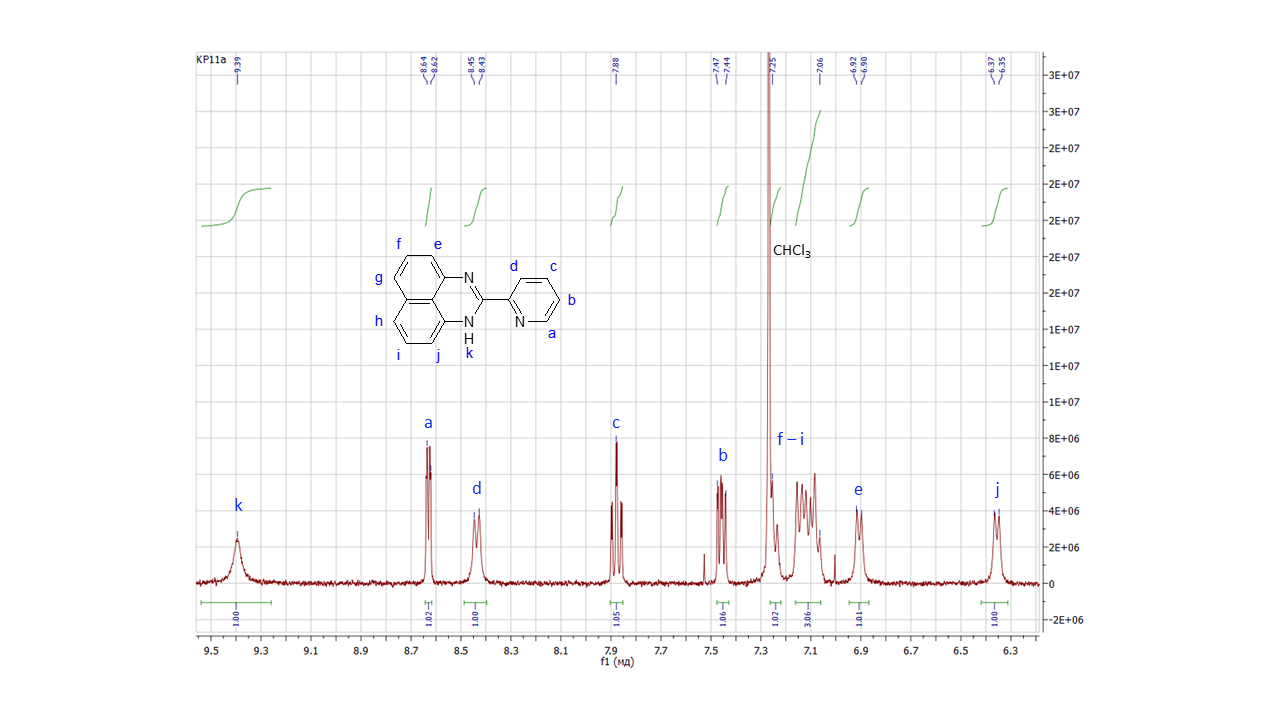

Supplement: Supplementary file 12 [file e-77-00096-sup12.tif]

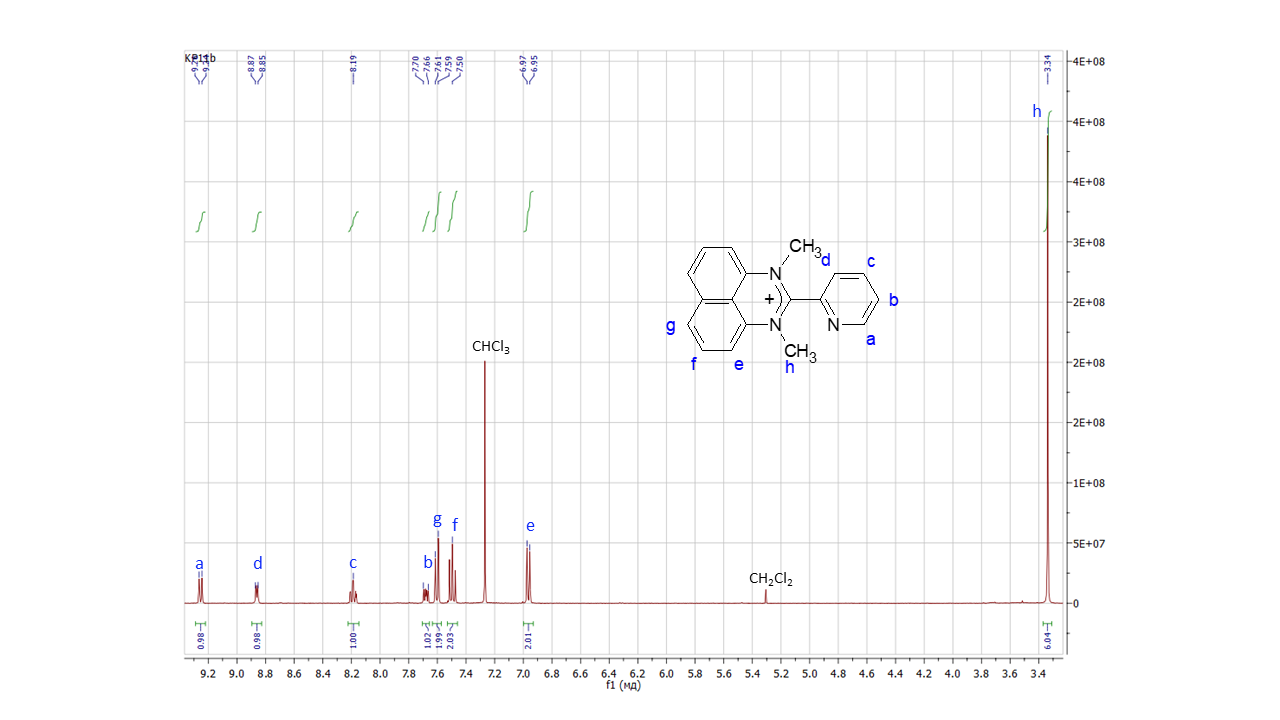

Supplement: Supplementary file 13 [file e-77-00096-sup13.tif]

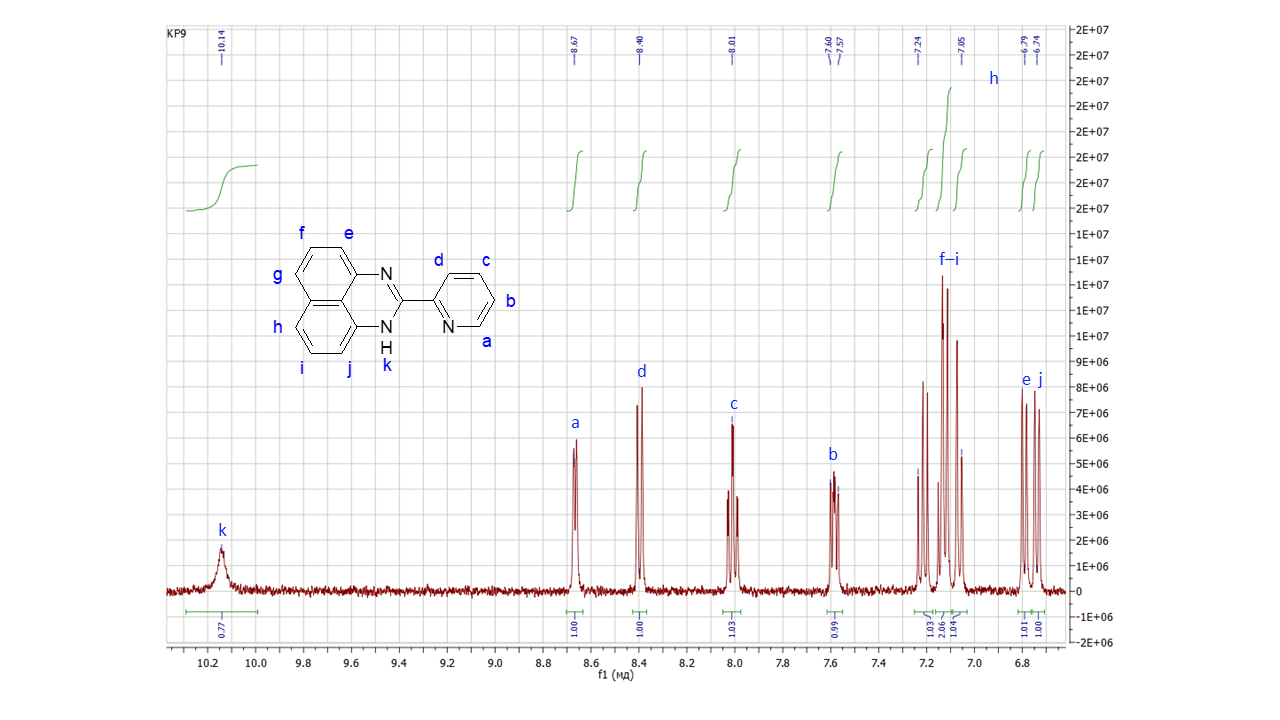

Supplement: Supplementary file 14 [file e-77-00096-sup14.tif]
